# Lumbar Level Peripheral Nerve Stimulation for Low Back Pain

**DOI:** 10.31486/toj.21.0115

**Published:** 2022

**Authors:** Kenneth J. Fiala, Roy B. Kim, Joshua M. Martens, Alaa Abd-Elsayed

**Affiliations:** ^1^ University of Wisconsin-Madison School of Medicine and Public Health, Madison, WI; ^2^ Department of Anesthesiology, University of Wisconsin-Madison School of Medicine and Public Health, Madison, WI

**Keywords:** *Chronic pain*, *electric stimulation*, *electrodes–implanted*, *low back pain*, *lumbosacral region*, *pain management*

## Abstract

**Background:** Low back pain affects the lives of millions of people in the United States and the world. Not only does low back pain affect the quality of life for the individual patient, but it also accounts for many emergency department and health care visits. For a subset of patients, conservative measures such as medications and physical therapy, nonsurgical interventions, and surgery are not effective. Peripheral nerve stimulation is an emerging treatment option for patients with chronic low back pain. This case series assesses 6 patients’ experiences with lumbar level peripheral nerve stimulation.

**Case Report:** Three male and 3 female patients underwent lumbar level peripheral nerve stimulation as a treatment for chronic low back pain. The average age of the patients was 63.5 years, and they demonstrated an average pain reduction of 64.8%.

**Conclusion:** This series provides evidence that lumbar level peripheral nerve stimulation may be an efficacious treatment for chronic low back pain that is refractory to conservative measures. Large studies are needed to assess the outcomes and durations of improvement associated with this treatment.

## INTRODUCTION

Low back pain affects millions of people across the globe, increasing in prevalence with age and peaking between the ages of 80 to 89 years with a higher incidence in females.^1^ Low back pain accounts for approximately 3.15% of all emergency department visits,^2^ and approximately 1 in 5 people who suffer from acute lower back pain will have unresolved symptoms at 1 year. Chronic back pain is described as pain that persists for 12 or more weeks.^3^ A systematic review of 9 studies assessing the prevalence of chronic low back pain found the prevalence to be between 3.9% and 20.3%.^4-13^

Medications, physical therapy, nonsurgical interventions, and surgery are treatment options for low back pain. Minimally invasive interventions include exercise, acupuncture, functional restoration, progressive relaxation, self-care options, traction, spa therapy, and massage.^14^ Nonsteroidal anti-inflammatory drugs, acetaminophen, skeletal muscle relaxants, acupuncture, superficial heat, physiological therapy, interdisciplinary rehabilitation, exercise therapy, spinal manipulation, opioids, brief individualized educational interventions, benzodiazepines, massage, and yoga have all been found to have a moderate net benefit.^14^

For patients refractory to these treatment options, radio-frequency ablation, surgery, and permanently implanted neuromodulation systems are used to treat chronic low back pain.^15^ While these more invasive treatment modalities may be a patient's only remaining recourse for intractable low back pain, complications associated with each option must be considered. Failed back surgery syndrome is a common complication of lumbar surgeries in which patients experience persistent, even aggravated, low back pain following surgical intervention.^16^ Peripheral nerve stimulation (PNS) offers an appealing alternative to these more invasive treatment modalities because the procedure is safe, minimally invasive, and reversible.^16^

In PNS, small electrodes are placed next to a peripheral nerve. Electrical pulses delivered through the electrodes stimulate the desired nerve for 60 days and then the electrodes and PNS device are removed. For some patients with chronic low back pain, PNS has been shown to provide significant improvements in patient quality of life and pain reduction lasting at least 12 months.^17^ Multiple prospective studies have been conducted to assess the effectiveness of PNS in patients with intractable low back pain, and all have found the procedure to be safe and effective in improving both pain and quality of life.^17-20^

Because impaired quality of life is one of the most prevalent negative impacts of chronic back pain,^21,22^ the results of these studies suggest that PNS should be considered as an intervention for these patients more often and perhaps earlier in their course of treatment. Studies by Kumar et al^23^ and van Gorp et al^24^ found that intervention with neuromodulative treatment, such as PNS, is associated with greater likelihood of pain relief and greater improvement in general health status overall. Despite the promising evidence surrounding PNS for use in patients with chronic low back pain, the procedure has associated complications, including lead migration, skin erosion, wound infection, dural puncture, and neurologic injury.^25^ Complication rates across studies range from 9% to 20% of patients.^17,20^ Ongoing investigation of these complications is critical to provide further evidence of the safety of PNS.

## METHODS

This case series underwent institutional review board review and received an exemption. All patients who underwent PNS implant met our defined criteria for chronic low back pain refractory to conservative management. Patients were considered eligible for intervention if they experienced nonspecific low back pain for at least 5 days per week for more than 2 months and did not experience relief following conservative management with a combination of pharmacotherapy, physical therapy, transcutaneous electrical nerve stimulation (TENS) units, chiropractics, sacroiliac joint injections, radiofrequency ablation, heat and ice, and rest. Prior to intervention, serious etiologies of low back pain, such as cauda equina syndrome or cancer, were ruled out.

For each of the 6 cases presented in this series, the approach described by Deer et al^15^ was used to place the SPRINT PNS System (SPR Therapeutics, Inc). Patients underwent fluoroscopy-guided percutaneous lead placement targeting the medial branch nerves at the lumbar levels at the center of the painful region. Correct placement was determined through ultrasound visualization of activation of the lumbar multifidi, and the leads were secured with surgical glue. The leads were then connected to wearable stimulators.

## CASE SERIES

### Case 1

A 66-year-old male with a history of hypertension, obesity, degenerative joint disease of the lumbar spine, and depression presented to the interventional pain clinic with a 30-year history of left-sided low back pain. The patient had a history of multiple accidents, including falling off a ladder that resulted in multiple fractures. The patient rated his pain at 3/10 on the visual analog scale (VAS) and described it as a constant “achy” pain that worsened with movement and had been worsening during the prior few years. The patient experienced minimal relief with pharmacotherapy and conservative management.

**Figure 1.**
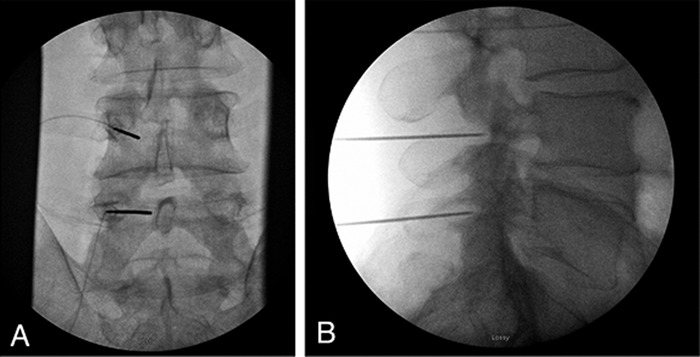
(A) Anterior posterior and (B) lateral radiographic images of the introducer placement for the SPRINT peripheral nerve stimulation device at the level of L4 and L5 on the left side, targeting the median nerves for the patient described in case 1.

The patient underwent implantation of a SPRINT peripheral nerve stimulator targeting the left median nerves at the L4 and L5 levels (Figure 1). The patient tolerated the procedure well and was able to ambulate out of the procedure area in the same fashion in which he arrived. The patient reported substantial improvement in his pain following the procedure, as well as improved sleep secondary to pain relief immediately following the procedure. Ten days following the procedure, the patient reported approximately 50% pain relief although he reported a fracture in 1 of the leads. Later in the course of treatment, the patient fell while performing construction work, and the single remaining PNS lead malfunctioned. The patient was scheduled for a SPRINT PNS System lead replacement as he found the device helpful when it was functional.

### Case 2

A 63-year-old male with a history of hypertension, hypogonadism, foraminal stenosis of the lumbar spine, obesity, and bilateral sciatica presented to the interventional pain clinic with progressing chronic low back and buttock pain following a lumbar laminectomy in 2019. He rated his right low back pain at 9/10 on the VAS and described it as a constant “sharp” pain radiating through the right thigh. The patient experienced minimal relief with pharmacotherapy, physical therapy, L4-L5 laminectomy, left facetectomy with interbody fusion, and C2-C7 cervical fusion.

**Figure 2.**
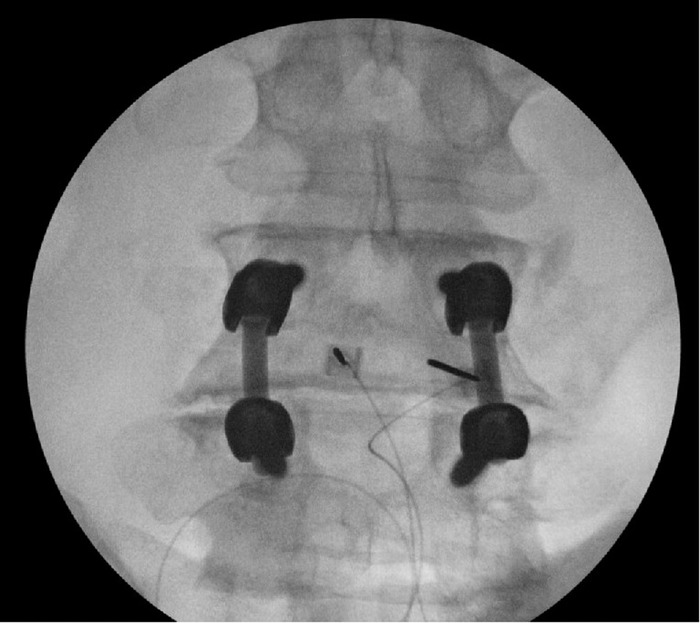
Radiographic image of the introducer placement for the SPRINT peripheral nerve stimulation device at the level of L4 bilaterally, targeting the median nerves for the patient described in case 2.

The patient underwent implantation of a SPRINT peripheral nerve stimulator bilaterally at the level of L4 (Figure 2). Eleven days after the procedure, the patient reported an improvement in pain to 5-6/10 on the VAS. He further noted that pain had improved to an aching pain, as opposed to a sharp pain, and it no longer radiated through his thigh. The patient also experienced an improvement in daily function, reporting that he was able to tolerate standing for longer periods of time than prior to the procedure. Eighteen days after the procedure, the patient accidentally pulled out a lead from the PNS, disconnecting it from the controller entirely. Following this incident, the patient's pain increased to 8/10 on the VAS and once again became sharp. The remaining electrode lead and PNS device were removed at this time. The patient did not return for follow-up.

### Case 3

A 66-year-old female with a history of hypertension, obesity, anxiety, stage IIIC high grade serous ovarian carcinoma, breast cancer, and osteopenia presented to the interventional pain clinic with chronic low back pain and foot/ankle/calf chemotherapy-induced peripheral neuropathy. She rated her pain as 6/10 on the VAS and described the pain as a band across her low back that was occasionally sharp and constantly “achy.” The patient experienced minimal relief with rest, Salonpas Pain Relief Patches (Hisamitsu America Inc.), TENS unit, pharmacotherapy (except gabapentin which provided some relief), physical therapy, chiropractic intervention, acupuncture, sacroiliac joint injections, and lumbar interspinous injection at L3-L4 and L4-L5.

**Figure 3.**
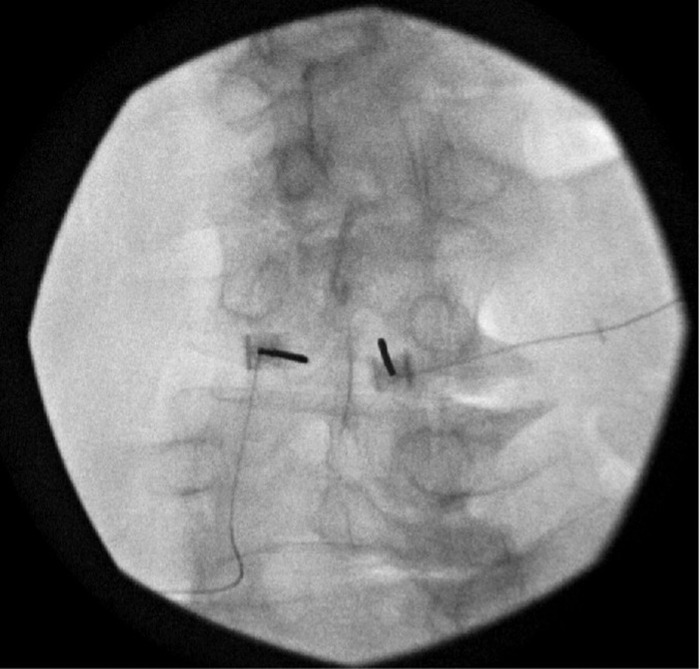
Radiographic image of the introducer placement for the SPRINT peripheral nerve stimulation device at the level of L4 bilaterally, targeting the median nerves for the patient described in case 3.

The patient underwent implant of a SPRINT peripheral nerve stimulator bilaterally at the level of L4 (Figure 3). Ten days after the procedure, the patient's pain had improved to 4/10 on the VAS. Her best report was 0/10, but her worst report was 8/10. The pain became intermittent and continued to improve with rest. The patient reported the negative effect of abnormally awakening at night following the PNS implant. Sixty-three days after the procedure, the patient's pain had improved further, decreasing to 3/10 on the VAS, and she reported improvements to her functional abilities. The patient did not report any increase in pain after the follow-up on day 63 postimplant.

### Case 4

A 70-year-old female with a history of hypertension, nerve entrapment syndrome, osteopenia, and degenerative joint disease of the lumbar region presented to the interventional pain clinic with chronic “achy” axial back pain that was worse on the right. The patient rated her pain as 10/10 when she presented to the clinic, although historically the pain was intermittent, improving to 0/10 at its best. The pain improved with rest and worsened with twisting, bending, sweeping, and vacuuming. The patient experienced minimal relief with pharmacotherapy, steroid injections, and lumbar medial branch radiofrequency ablation.

**Figure 4.**
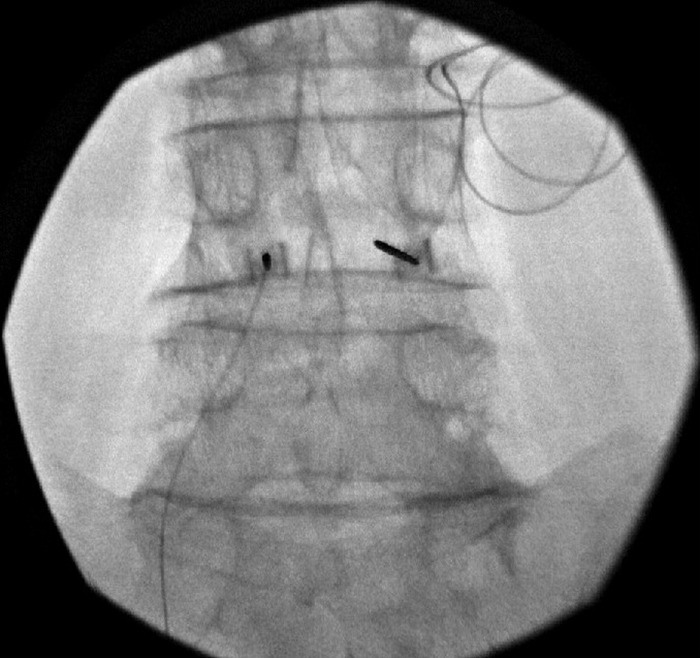
Radiographic image of the introducer placement for the SPRINT peripheral nerve stimulation device at the level of L4 bilaterally, targeting the median nerves for the patient described in case 4.

The patient underwent implant of a SPRINT peripheral nerve stimulator bilaterally at the level of L4 (Figure 4). Eleven days after implant placement, the patient reported 100% pain relief that was immediate. Negative effects from the PNS implant included a mild rash at the dressing site and a light feeling of pressure from the implant that the patient said did not bother her. Fifty-three days after implant placement, the patient continued to report 100% pain relief and noted great satisfaction with her PNS device. She had the PNS device removed at this time, with plans for additional follow-up. Eighty-eight days after implant placement and 35 days after device removal, her pain was 4/10 on the VAS (7/10 at the worst and 0/10 at the best). She described the pain as constant and aching with improvement during daytime hours but worsening with lying in bed, vacuuming, and lifting. The patient did not report any increase in pain after the follow-up on day 88 postimplant.

### Case 5

A 48-year-old male with a history of hypertension, morbid obesity, and osteoarthritis presented to the interventional pain clinic with chronic bilateral low thoracic back pain resulting from a vehicular accident. The patient rated his pain as 6/10 on the VAS and described it as sharp and tight, with the location around the ribcage bilaterally and the feeling “like I am doing sit-ups.” The pain was constant but worsened with sitting too long and twisting. Pain was improved by lying down. The patient experienced minimal relief with pharmacotherapy.

**Figure 5.**
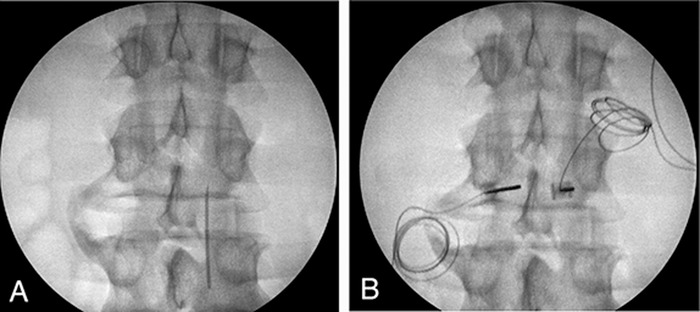
**(A) Radiographic image identifying target placement for the introducer with a pointer for the SPRINT peripheral nerve stimulation device at the level of L3, targeting the median nerves for the patient described in case 5.** (**B) Radiographic image of the introducer placement for the SPRINT peripheral nerve stimulation device at the level of L3 bilaterally, targeting the median nerves for the patient described in case 5.**

The patient underwent bilateral L3 SPRINT peripheral nerve stimulator implant (Figure 5). The patient reported great relief following the implant and said he was able to use his back for 9 hours each day postprocedure. The patient still reported some pain with twisting or sitting for a long time. The patient's leads fell out approximately 60 days postprocedure. He missed follow-up appointments but reported by phone that he experienced 80% to 90% ongoing relief 123 days after the procedure. The patient has not reported a change in pain since the follow-up 123 days postimplant.

### Case 6

A 68-year-old female with a history of hypertension, obstructive sleep apnea, and anxiety presented to the interventional pain clinic with a 3-year history of chronic low back pain. Her pain was axial; she described it as burning and sharp and rated it at a constant 6-10/10 on the VAS with a band-like shape in her lower back with no referral pattern. The patient experienced minimal relief with pharmacotherapy, dry needling, physical therapy, and L4-S1 bilateral radiofrequency ablation. The patient found some relief with a sacroiliac joint injection, lumbar trigger point injections, and bilateral greater trochanteric bursa injections.

**Figure 6.**
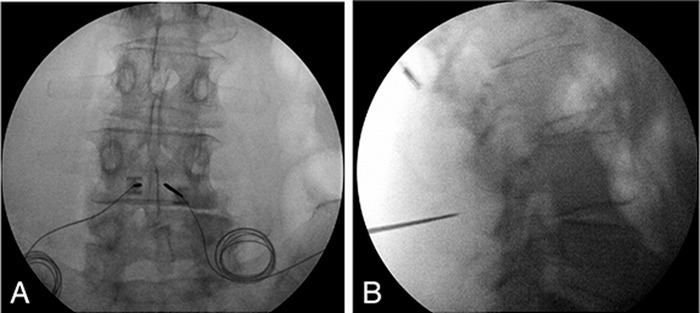
(A) Anterior posterior and (B) lateral radiographic images of the introducer placement for the SPRINT peripheral nerve stimulation device at the level of L4 bilaterally, targeting the median nerves for the patient described in case 6.

The patient underwent bilateral L4 SPRINT peripheral nerve stimulator lead placement (Figure 6). Eleven days following the implant, the patient reported “100% pain relief.” Initially she felt sensation in her abdomen because her stimulation was too high, but at the 11-day follow-up, she denied any paresthesias after adjustment of the stimulation parameters. At that time, the patient denied any other side effects or issues other than some difficulty changing her dressings. Sixty days following implant, the patient reported she was still doing very well and described the PNS device as a “miracle.” The patient experienced some troubleshooting issues with the device initially but otherwise denied any issues or side effects. During the 60-day follow-up, the PNS device was removed. The latest follow-up was 426 days postimplant, and the patient still reported 100% relief. The patient has not reported a change in pain since this follow-up.

## DISCUSSION

The 6 cases are summarized in the Table. The patients in cases 4 and 6 reported preprocedure pain scores that varied in severity, so the highest pain scores reported were used to calculate the percentage reduction in pain score. The patients in cases 1, 5, and 6 did not report a postprocedure pain score but instead reported a postprocedure percent reduction in pain. These percent reductions were calculated into a postprocedure pain score from their preprocedure pain score. The patient in case 2 experienced an accidental removal of leads early in treatment, so the postprocedure pain score reported when the device was still in place was used. The patient in case 4 reported varying postprocedure pain scores from when the pain was at its best and at its worst, so these 2 scores were averaged to calculate a postprocedure pain score. For the patient in case 5, the average percent reduction in pain was used for the calculation.

**Table.** Patient Demographics, Baseline Information, and Pain Scores

Patient	Age, Years	Sex	BMI, kg/m^2^	Imaging Findings	Level of PNS	Preprocedure Pain Score	Postprocedure Pain Score	% Reduction in Pain Score
1	66	M	29.5	Bilateral facet arthropathy L1-L5, mild broad-based disc bulge L2-L3, L3-L4, and L4-L5	Left L4-L5	3	1.5	50
2	63	M	33	Prior intervention, likely degenerative minimal retrolisthesis of L2 on L3, degenerative disc changes, disc space narrowing and marginal osteophyte formation most pronounced at L5-S1, facet osteoarthritis of lower lumbar spine, mild anterior height loss of T12	Bilateral L4	9	5.5	39
3	66	F	37.8	Degenerative disc disease, mild foraminal stenosis on the left L4-L5, moderate right foraminal stenosis at L5-S1	Bilateral L4	6	3	50
4	70	F	30.6	Grade 1 retrolisthesis of T12 on L1, grade 1 anterolisthesis of L4 on L5 secondary to facet degenerative changes, moderate to severe degenerative disc disease and facet arthropathy that is most severe at L4-L5	Bilateral L4	10	3.5	65
5	48	M	40.2	Trace retrolisthesis of L4-L5, osseous fusion across the bilateral sacroiliac joints, mild disc desiccation at T-11-T12, L3-L4, L4-L5, and L5-S1, and disc edema at L5-S1	Bilateral L3	6	0.9	85
6	68	F	40.7	Progressive multilevel degenerative disc disease and hypertrophic facet arthropathy involving the lumbar and lower thoracic spine regions, with associated thoracolumbar scoliosis	Bilateral L4	10	0	100
Average	63.5	–	35.3	–	–	7.3	2.4	64.8

Note: Pain scores are based on the visual analog scale, with 0 signifying no pain and 10 signifying the worst possible pain.

BMI, body mass index; F, female; M, male; PNS, peripheral nerve stimulation.

All of the patients presented in this series reported a decrease in pain scores with implantation of a PNS device. The average percent pain reduction among the 6 patients was approximately 64.8%, equating to a mean pain score drop of 4.9. Two patients had better pain relief with the device in place. One of the 6 procedures targeted L3 bilaterally, 4 of the 6 targeted L4 bilaterally, and 1 targeted both L4 and L5 on the left side.

All 6 patients—3 males and 3 females—presented with a medical history including hypertension, 5 of 6 were obese (body mass index >30 kg/m^2^), and 2 of the 6 presented with a history significant for anxiety. The average age for the 6 patients was 63.5 years, with an age range of 48 to 70 years. These cases provide data on potential variables for predicting the efficacy of PNS in treating back pain.

As with any procedure, identifying the ideal patient population to undergo PNS for chronic low back pain is essential. Evidence suggests that younger patients have improved pain reduction as a result of PNS vs older patients.^26^ Additionally, randomized controlled trials conducted by van Gorp et al reported that the only other factor associated with effectiveness of PNS was time since onset of pain, finding that quicker intervention with PNS showed greater likelihood of pain relief.^24^ Future clinical trials should investigate the impact of obesity, hypertension, and other chronic conditions on the efficacy of PNS. Patients whose pain is directly impacted by a chronic condition such as obesity might experience a difference in benefit from PNS intervention vs patients without chronic conditions.

Because lumbar level PNS is a relatively new procedure, all negative side effects need to be documented in the literature. Negative observations from these 6 patients following PNS placement included abnormally awakening at night, a mild rash at the dressing site, difficulty changing dressings, accidental removal of leads, and some pressure at the site of implantation. The literature on PNS use in patients with low back pain does not include investigation of the etiology of such negative effects, and although these negative side effects are minor, further investigation of them in clinical studies is important to confirm PNS as a safe option for patients. Positive effects other than pain reduction reported by the patients in this series were improved sleep, the ability to stand for longer periods without pain, and functional improvement.

The mechanism of pain relief provided by PNS remains poorly understood. The gate control theory postulates that the pain relief provided by neuromodulation interventions arises from the inhibition of A-delta and unmyelinated C fibers via subcutaneous stimulation of myelinated A-beta fibers within the spinal cord by devices such as peripheral nerve stimulators.^27^ With PNS implantation for low back pain, not only are medial branch nerves targeted, but the intrinsic back muscle—the multifidus—is also stimulated. Wallwork et al showed that people with chronic low back pain have smaller multifidus muscles compared to healthy individuals.^28^ The placement of the PNS lead is typically in the substance of the multifidus muscle and to identify correct placement, stimulation resulting in selective unilateral multifidus activation is visualized on ultrasound.^29^

Some patients do not experience effective pain relief from PNS. In their study regarding the safety and efficacy of PNS, Ishak et al hypothesized that patients with a history of large skin incisions and multilevel surgical preparations may not benefit from PNS because of irritation of subcutaneous nerves that modifies the ability of terminal sensory afferent nerves to depolarize in the presence of the electrical field generated by PNS devices.^16^ While all patients included in this case series experienced pain relief from PNS placement, future clinical studies should investigate the patient history and demographic factors associated with decreased efficacy of pain reduction via PNS along with analyzing the duration of its effect.

## CONCLUSION

Patients with refractory chronic low back pain may find pain relief and functional improvement with PNS of the medial branch nerves at the lumbar spine level. This 6-case series suggests the potential for not only a large reduction in pain but also an increase in daily living function. These 6 patients showed an average pain reduction of 64.8% with minimal side effects. Further research needs to be conducted to assess the efficacy of using PNS as a treatment for chronic lower back pain in a larger population, the severity of less common complications, and the impact that variables such as sex and obesity have on the effectiveness of PNS. Additionally, further research should be conducted to improve this procedural technique to maximize its effectiveness, minimize any complications, and assess the duration of its potential relief.

## Notes

*This article meets the Accreditation Council for Graduate Medical Education and the American Board of Medical Specialties Maintenance of Certification competencies for Patient Care and Medical Knowledge.*
